# Energy Matching in NV Spin‐Phonon Interaction Enabling High‐Temperature Quantum Sensing via Lifetime Readout

**DOI:** 10.1002/advs.78645

**Published:** 2026-11-10

**Authors:** Yujing Cao, Junjie Lin, Wei Wang, Qianru Chen, Yan Liu, Jie Ren, E Wu, Heping Zeng

**Affiliations:** ^1^ State Key Laboratory of Precision Spectroscopy East China Normal University Shanghai China; ^2^ Beijing Academy of Quantum Information Sciences Beijing China; ^3^ Center For Phononics and Thermal Energy Science China‐EU Joint Lab on Nanophononics School of Physics Science and Engineering Tongji University Shanghai China; ^4^ Shanghai Key Laboratory of Magnetic Resonance Institute of Magnetic Resonance and Molecular Imaging in Medicine School of Physics East China Normal University Shanghai China; ^5^ Chongqing Key Laboratory of Precision Optics Chongqing Institute of East China Normal University Chongqing China; ^6^ Collaborative Innovation Center of Extreme Optics Shanxi University Taiyuan Shanxi China

**Keywords:** excited‐state lifetime, nitrogen‐vacancy center, optically detected magnetic resonance, quantum sensing, spin‐phonon interaction

## Abstract

High‐temperature quantum sensing with NV centers has long been hindered by the spin contrast degradation from the thermally accelerated intersystem crossing (ISC) and the thermally induced fluorescence instability in the conventional intensity‐based readout. Here, we systematically investigate the NV spin‐phonon interaction in the ISC transition and the zero‐field splitting (ZFS) shift, which are two key aspects governing NV high‐temperature sensing performance. We report an energy matching between their characteristic phonon modes, which enables the ISC‐induced sensitivity degradation to be effectively compensated by the steepened slope of the ZFS shift, thereby preserving a high sensitivity even at high temperatures. This effect is particularly evident in the lifetime‐based optically detected magnetic resonance (ODMR) measurements, as the lifetime readout remains robust against thermally induced fluorescence instability at high temperatures. We reach a shot‐noise‐limited temperature sensitivity of 8.3 µK Hz^−1/2^ at 490 K in the lifetime‐based continuous‐wave ODMR scheme. Our work has opened up opportunities for highly sensitive NV quantum sensors under extreme conditions.

## Graphical abstract

Quantum sensing at high temperatures is challenging for the NV center. We overcome this by revealing an energy matching within NV spin‐phonon interactions. This match preserves high sensitivity upon heating. By reading out the NV excited state lifetime, we demonstrate a shot‐noise‐limited sensitivity of 8.3 µK Hz^−^^1^^/^^2^ at 490 K, paving the way for robust quantum sensing under extreme conditions.

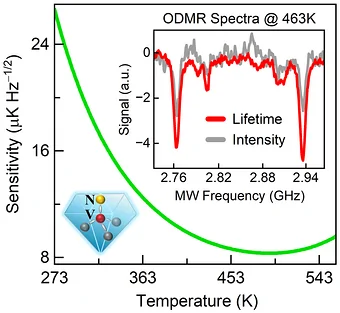

## 1 Introduction

The negatively charged nitrogen‐vacancy (NV) center in diamond has become a leading platform for quantum sensing due to its bright fluorescence emission, convenient optical spin readout capability [1], and exceptional sensitivity to various physical quantities including magnetic field [2, 3, 4], electric field [5], temperature [6, 7, 8, 9], gravity [10], and pressure [11]. Despite these advantages, high‐temperature operation has long been a recognized weakness of NV‐based quantum sensing because the thermally accelerated intersystem crossing (ISC) transition progressively degrades spin contrast, and the conventional readout of fluorescence intensity suffers from severe fluorescence instability upon heating [7, 8]. These obstacles become particularly acute at elevated temperatures, hindering NV sensors from achieving high‐sensitivity quantum metrology.

Recent advances have been made in lifetime‐based quantum metrology using NV sensors, exploiting the robustness of lifetime readout against technical noise including sample drifting and excitation laser power fluctuations [12, 13, 14, 15, 16, 17]. Our previous work has demonstrated that the lifetime‐based magnetometry surpasses the conventional intensity‐based method in the long‐term sensing precision at room temperature, and also performs well in complex environments such as biological solutions [15, 16, 17]. Compared to the conventional intensity readout that only reflects the steady‐state excited‐state (ES) population, the lifetime readout method can directly capture the population reduction dynamics of ES [18, 19, 20], enabling accurate characterization of the fundamental spin‐phonon interaction, which has yet to be fully addressed both theoretically and experimentally. Therefore, the lifetime readout offers a promising route for a high‐performance NV sensor at high temperatures.

Specifically, the spin‐phonon interaction of the NV center governs two critical processes in its sensing performance, which are the ISC transition and the energy level shift of the zero‐field splitting (ZFS). On the one hand, as a phonon‐assisted transition over an energy barrier, the ISC rate can be accelerated through phonon coupling, thereby modulating the spin contrast. On the other hand, the ZFS shift originates from the phonon‐mediated spin‐lattice interaction, whose thermal response is a key factor in the temperature sensitivity. Extensive studies have been conducted on each aspect individually, such as the calculation of electronic structures [21, 22, 23] and the characterization of defect‐lattice interaction [24, 25, 26]. However, the correlation between the phonon modes governing the ISC dynamics and the ZFS shift has yet to be explored, leaving a critical gap in describing the high‐temperature performance of NV sensors.

Here, we theoretically and experimentally investigated the NV spin‐phonon interaction over a wide temperature range, focusing on the competitive influence between the ISC transition and the ZFS shift on the high‐temperature sensing performance. We report an energy matching in the characteristic phonon modes governing these two processes. At high temperatures, thanks to the thermal occupation of phonon modes with the matched energy, the ISC‐induced sensitivity degradation can be effectively compensated by the simultaneously steepened slope of the ZFS shift. Exploiting the robustness of lifetime readout against the thermally induced fluorescence instability, we experimentally demonstrated that the lifetime‐based sensing performance is preserved by this energy matching at high temperatures. By conducting continuous‐wave optically detected magnetic resonance (cw‐ODMR) measurements on the NV ensemble, we can reach a shot‐noise‐limited temperature sensitivity of 8.3 µK Hz^−1/2^ at 490 K via lifetime readout. Our work provides a benchmark for lifetime‐based high‐temperature NV quantum sensing. The lifetime readout can also be extended to other solid‐state quantum platforms, providing a versatile tool for analyzing phonon‐mediated spin dynamics and optimizing sensing performance.

## 2 Results and Discussion

### 2.1 Spin‐Phonon Interaction Model

Figure 1a shows the simplified NV energy structure. *D*(*T*) is the temperature‐dependent ZFS of the NV ground state (GS). Under an external magnetic field *B* along the NV symmetry axis, the GS *m_s_* = ± 1 levels exhibit Zeeman splitting, with an energy separation of 2γ*B*, where γ = 28.024 MHz mT^−1^ is the NV gyromagnetic ratio [27]. Upon green laser excitation, the direct decay from the spin‐triplet ES to GS is radiative and spin‐conserving. Meanwhile, the ISC processes from the ES to the metastable singlet state and then to the GS are non‐radiative transitions, where the ES *m_s_* = ± 1 states have a higher probability of decaying back to the GS *m_s_* = 0 state. This behavior arises from different coupling mechanisms: the ES *m_s_* = ± 1 states decay to the singlet state via the first‐order direct spin‐orbit coupling, while the ES *m_s_* = 0 state can only decay through the second‐order phonon‐assisted process [28]. Therefore, at low temperatures, the ISC transition rates of the *m_s_* = ± 1 states are faster than that of the *m_s_* = 0 state, resulting in reduced fluorescence intensity and shortened ES lifetime (Figure 1b). As the temperature increases, the occupation of high‐energy phonons augments, promoting phonon‐assisted transitions over the energy barrier, thereby accelerating the ISC rates. This spin‐phonon interaction can be evaluated by the temperature dependence of the ES lifetime *τ_ms_* (*T*) for each spin state *m_s_*, which is described by the Mott‐Seitz formula through multi‐phonon emission [21],

(1) $$\tau_{ms}\left(T\right)=\frac{\tau_{ms}\left(T_{0}\right)}{1+A_{ms}\exp\left[-\frac{\Delta E_{ms}}{\kappa_{B}T}\right]},$$

where *T*_0_ is the initial temperature, κ_B_ is the Boltzmann constant, *A_ms_* is the coupling coefficient describing the strength of coupling to the phonon bath, and *∆E_ms_* is the energy barrier of the ISC processes. The corresponding temperature‐dependent ISC rate is determined by [21]

(2) $$\Gamma_{ISC}\left(T\right)=\frac{1}{\tau \left(T\right)}-\frac{1}{\tau_{rad}},$$

where 1/*τ*_rad_ is the radiative decay rate that is independent of spin and temperature. Specifically, *∆E_ms_*_= 0_ is the energy barrier for a pure phonon‐assisted ISC transition. At high temperatures, the enhanced occupation of the characteristic phonon modes will significantly accelerate *Γ*_ISC_ of the *m_s_* = 0 state, leading to spin mixing and limiting the spin contrast.

**FIGURE 1:**
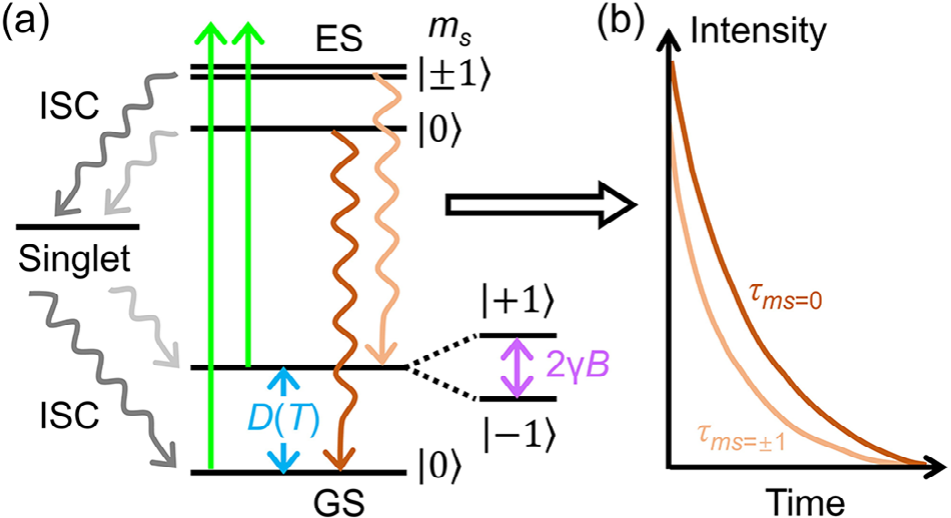
(a) Level diagram of the NV center, where *D*(*T*) is the temperature‐dependent ZFS. The GS *m_s_* = ± 1 levels experience a Zeeman shift in the presence of a magnetic field *B*. The laser excitation is represented by green arrows, the phonon‐mediated fluorescence emission by orange arrows, and the phonon‐mediated ISC processes by grey arrows. (b) Illustration of fluorescence decay curves from ES to GS for the *m_s_* = 0 and *m_s_* = ± 1 states, respectively.

Another key aspect of the NV spin‐phonon interaction is the temperature‐dependent ZFS shift *D*(*T*), which originates from thermally induced atom displacements in the lattice [24, 28]. To accurately describe its phonon occupation, we adopted the two‐phonon‐mode model proposed in Reference 24, which describes the ZFS shift as proportional to the occupancy numbers of two representative phonon modes,

(3) $$D\left(T\right)=D_{0}+c_{1}n_{1}+c_{2}n_{2},$$

where *D*_0_ is the ZFS at 0 K and *c*_i_ is the coupling coefficient of the i‐th phonon mode. The occupant number *n*_i_ follows

(4) $$n_{i}=\frac{1}{\exp\left[\frac{\Delta_{i}}{\kappa_{B}T}\right]-1},$$

where *∆*_i_ is the characteristic mode energy.

Using this model, we evaluated the characteristic mode energy of *D*(*T*) according to the experimental data of single‐NV in Reference 21, and obtained *∆*_1_≈67.1 meV and *∆*_2_≈116.6 meV. The characteristic lattice relaxation energy was estimated through *S·∆*_i_ [29, 30], with its Huang‐Rhys factor *S*≈3.65 for single‐NV [21]. We found that the characteristic lattice relaxation energy of the second phonon mode is about 426 meV, close to the phonon‐assisted ISC energy barrier *∆E_ms_*_= 0_≈480 meV in the same experiment. Such proximity indicates the existence of energy matching between these two phenomena, where the occupation of phonon modes simultaneously modulates the ZFS shift and ISC transition rate. Consequently, as the temperature increases, the temperature sensitivity improves by *dD*(*T*)/*dT* [31], while being counteracted by the enhanced spin mixing caused by the accelerated *Γ*_ISC_ of the *m_s_* = 0 state. If this competitive influence between thermally enhanced ZFS shift and the thermally reduced spin contrast is appropriately utilized, the high‐temperature sensitivity of NV sensors can be well preserved. This phenomenon is expected to be more pronounced in an NV ensemble than in individual NV centers, as higher NV concentrations will reduce *∆E_ms_*_= 0_ and enhance the spin‐phonon coupling [32].

### 2.2 Spin‐Phonon Interaction of NV Ensemble

Inspired by this observation, we investigated the spin‐phonon interaction in the ES lifetime and ZFS shift of the NV ensemble at high temperatures. Figure 2a shows the experimental setup, in which a single‐crystal (111)‐oriented chemical vapor deposition (CVD) diamond ([N] ≈ 10 ppm) sample was used to study the temperature dependence of ES spin dynamics. The diamond is 3×3×0.2 mm^3^, double‐side polished with a surface roughness below 10 nm, and the NV centers were formed in situ during CVD growth. Owing to the (111)‐oriented growth, the NV ensemble exhibits preferential orientation along the [111] axis. The bottom surface of the diamond sample was adhered to a real‐time temperature controller (VAHEAT, Interherence GmbH). A permanent magnet was fixed near the sample to provide a static external magnetic field. Spin resonance was driven by microwaves (MW), which were transmitted through a coplanar circular microstrip antenna placed on the top surface of the sample. The above devices were combined and installed on a conventional manual 3D translation stage without the need for active position control. The fluorescence signal was collected only through a 4× achromatic objective lens and detected by a single‐photon detector. The excitation laser was a 532 nm picosecond pulse laser with a repetition rate of 20 MHz and an average power of 0.7 mW. Within the 50 ns laser pulse period, multiple excitations of the NV center could be avoided. The 12.5 ps pulse duration was significantly shorter than the NV's excited state lifetime. The fluorescence decay curve was measured with a time‐correlated single‐photon counting module, which records the arrival time of detected fluorescence photons relative to the falling edge of the excitation laser pulses, with a time‐bin of 128 ps.

**FIGURE 2:**
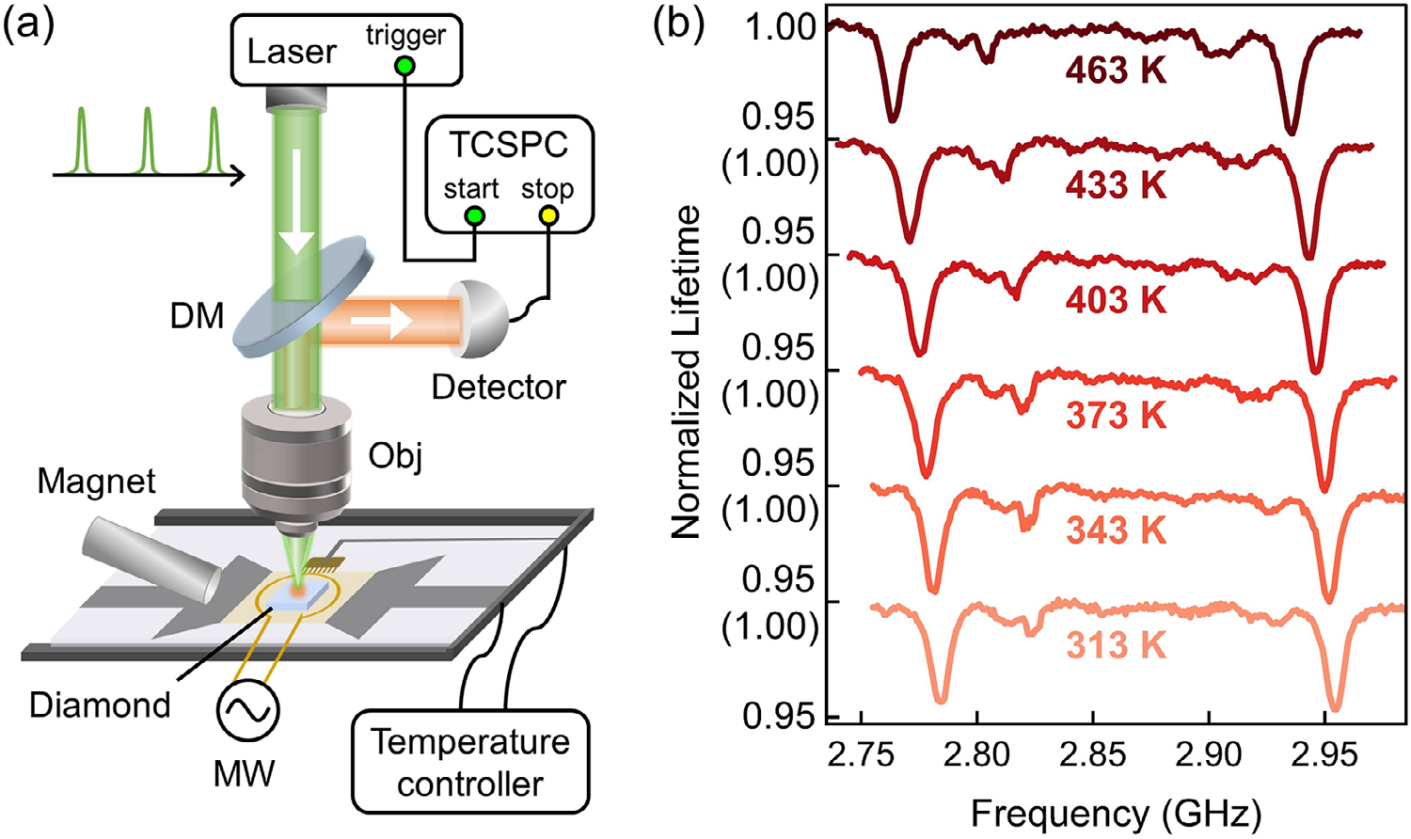
(a) Schematic of the experimental setup. The diamond sample was adhered to a resistance heating plate equipped with a resistive temperature detector for temperature control. The external magnetic field was generated by a magnet. MWs were delivered through a ring antenna on the sample surface. Fluorescence decay under 532 nm pulsed excitation was detected by a single‐photon detector and measured in a time‐correlated single‐photon counting module. (b) Temperature dependence of the lifetime‐based ODMR spectra, obtained by fitting the fluorescence decay curves at each MW frequency.

For the convenience of spin control and measurement, the cw‐ODMR scheme was adopted, in which laser excitation and MW field were applied simultaneously, and the resonant MW continuously drove GS spin from *m_s_* = 0 state to *m_s_* = ± 1 states [2]. Here, following the established definition in ODMR measurement protocols [2], “cw‐ODMR” denotes the simultaneous and continuous application of MW field and laser excitation without any additional gating, distinguishing it from pulsed ODMR protocols in which MW and laser are applied sequentially. Figure 2b shows the lifetime‐based ODMR spectra from 313 to 463 K, where the NV fluorescence decay curves were recorded at different MW frequencies and fitted using a single‐exponential function to obtain the ES lifetime values. In the conventional intensity readout, the contrast and the signal stability of ODMR rapidly decrease upon heating, mainly due to thermally induced effects such as fluorescence quenching, blinking, and charge‐state conversion [7, 8, 33]. In contrast, even at the highest temperature in our experiment of 463 K, the lifetime readout could maintain high‐quality ODMR spectra, demonstrating its exceptional robustness to thermal variation and system noise. This stability enables accurate characterization of the spin‐phonon interaction across the entire temperature range.

We first characterized the phonon‐assisted energy barriers for ISC transitions via temperature‐dependent ES lifetimes *τ_ms_* (*T*). *τ_ms_*_= 0_(*T*) was measured by continuously applying MW with an off‐resonant frequency at 2 GHz while tuning the sample temperature from 313 to 463 K. And *τ_ms_*_= ± 1_(*T*) was measured with the corresponding resonant frequency at different temperatures. The experimental data were then modeled using the Mott‐Seitz formula described in Equation (1) as shown in Figure 3a. The simulation temperature range was set from 210 to 560 K so that the ES orbital hopping and spin mixing, as well as the second‐order spin‐phonon interaction, could be ignored in the present model because the ES orbital hopping and spin mixing become significant only at low temperatures [34] and the second‐order spin‐phonon interaction only dominates at high temperatures [35]. The obtained energy barriers are *∆E_ms_*_= 0_ = 179.3 ± 9.0 meV, *∆E_ms_*_= −1_ = 125.0 ± 6.3 meV, and *∆E_ms_*_= +1_ = 107.4 ± 5.4 meV, with the corresponding coupling coefficients of *A_ms_*_= 0_ = 3.75 ± 0.02, *A_ms_*_= −1_ = 0.95 ± 0.04, and *A_ms_*_= +1_ = 0.80 ± 0.02. At low temperatures, the exponential term in Equation (1) is negligible for all spin states, and the lifetime remains roughly constant. As the temperature increases, due to the lower energy barrier of the *m_s_* = ± 1 states, *τ_ms_*_= ± 1_ starts to decrease at lower temperatures than *τ_ms_*_= 0_. However, their phonon coupling strength is weaker than that of the *m_s_* = 0 state, with *A_ms_*_= 0_ almost four times stronger than *A_ms_*_= ± 1_. Consequently, at high temperatures, the increase in the occupation of high‐energy phonons results in a faster decrease in *τ_ms_*_= 0_ than *τ_ms_*_= ± 1_. This behavior is more evident in the temperature dependence of ISC rates *Γ*_ISC_(*T*), which is calculated using Equation (2) and plotted in Figure 3c. Compared to that in the *m_s_* = ± 1 states, the increase of *Γ*_ISC_ in the *m_s_* = 0 state is slower at low temperatures, but exhibits greater acceleration at high temperatures. Theoretically, the second‐order phonon‐assisted ISC transition of the *m_s_* = 0 state is more sensitive to the occupation of high‐energy phonons than the first‐order direct spin‐orbit coupling of the *m_s_* = ± 1 states [28].

**FIGURE 3:**
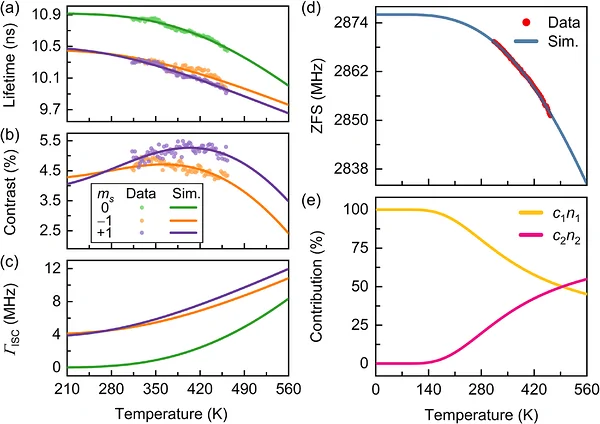
Experimental results and simulations of the *m_s_* = 0 (green), *m_s_* = −1 (orange), and *m_s_* = +1 (purple) states in the lifetime‐based ODMR measurements. Temperature dependence of (a) ES lifetime values, (b) spin contrast, (c) ISC rates, (d) ZFS, and (e) ZFS contributions from two characteristic phonon modes is plotted. The temperature range of simulation in a, b, c is 210–560 K, and in d, e is 0–560 K. Experimental data were measured from 313 K to 463 K.

Accordingly, the spin contrast defined as (1−*τ_ms_*_= ± 1_/*τ_ms_*_= 0_)×100% exhibits nonmonotonic temperature dependence (Figure 3b). The initial increase reflects that at low temperatures, the thermally enhanced *Γ*_ISC_ in the *m_s_* = 0 state is weaker than that in the *m_s_* = ± 1 states. At higher temperatures, due to the increase in the occupation of high‐energy phonons, the preferential acceleration of *Γ*_ISC_ in the *m_s_* = 0 state leads to a decrease. It is worth noting that the degradation of spin contrast in the lifetime readout is less than 6% throughout the entire experimental temperature range. However, due to the thermally induced fluorescence instability, the degradation in the intensity readout exceeds 30% in the same experiment. This remarkable robustness in the lifetime readout makes it a powerful tool for NV‐based sensing under extreme conditions.

Moreover, compared to the *m_s_* = −1 state, the *m_s_* = +1 state exhibits lower *∆E_ms_* and *A_ms_*_,_ resulting in higher spin contrast with less degradation during heating. This asymmetry may be due to the combined effects of the inhomogeneous NV distribution and orientation, the residual strain field, and the transverse magnetic field components [2]. It also emphasizes the importance of spin‐defect engineering and provides additional degrees of freedom for optimizing the performance of NV sensors.

We next address the spin‐phonon interaction of the ZFS shift *D*(*T*), which was experimentally determined from the lifetime‐based ODMR spectrum at each temperature. By fitting the experimental data to the two‐phonon‐mode model described in Equation (3) and (4) as shown in Figure 3d, we obtained the ZFS at 0 K to be *D*_0_ = 2876.0 ± 0.8 MHz, with the characteristic phonon mode energies *∆*_1_ = 70.0 ± 2.4 meV and *∆*_2_ = 155.5 ± 4.3 meV, as well as the corresponding coupling coefficients *c*_1_ = −61.4 ± 0.4 MHz and *c*_2_ = −550.0 ± 1.3 MHz. Compared with the reported values for single NVs [21, 24], the mode energies determined here are roughly consistent, while the coupling coefficients are higher, confirming that the phonon coupling strength in the NV ensemble is stronger.

According to Reference 30, using a Huang‐Rhys factor *S*≈2.6 for the CVD‐grown NV ensemble, we evaluated the characteristic lattice relaxation energies of the first and the second phonon modes corresponding to the ZFS shift, which are *S·∆*_1_ ≈182.0 meV and *S·∆*_2_ ≈404.3 meV, respectively. Energy matching between *S·∆*_1_ and *∆E_ms_*_= 0_ = 179.3 ± 9.0 meV could be observed. This energy matching is different from the single‐NV case previously discussed, where *∆E_ms_*_= 0_≈480 meV actually matches the relaxation energy of the second phonon mode *S·∆*_2_≈425 meV. Although *∆E_ms_*_= 0_ in the NV ensemble is relatively low, thermally activated ISC transitions require multi‐phonon absorption, which primarily depends on the number of phonons involved, thereby reducing the effect of the energy barrier height. Hence, the thermally induced degradation of spin contrast in the NV ensemble is not more severe than in single NVs [7, 8, 21]. Besides, the enhanced phonon coupling strength in the NV ensemble is favorable for improving the temperature sensitivity, as it makes the ZFS shift more sensitive to temperature variations. More importantly, in our ensemble sample, the almost perfect energy matching between *∆E_ms_*_= 0_ and *S·∆*_1_​ indicates that the increase of *dD*(*T*)/*dT* effectively compensates for the degradation of spin contrast. Consequently, precision sensing performance is preserved at high temperatures.

### 2.3 High‐Temperature Sensing via Lifetime Readout

We calculated the shot‐noise‐limited temperature sensitivity *η*_cw,T_ and magnetic field sensitivity *η*_cw,B_ using the simulation results in Figure 3. The shot‐noise‐limited temperature sensitivity *η*_cw,T_ is defined by [2, 31]

(5) $$\eta_{cw,T}=\frac{\;4\;\Delta \omega }{3\sqrt{3}\pi \frac{d\omega_{+}\left(T\right)}{dT}C_{cw}\;\sqrt{R_{max}}}\;,$$

where *∆ω* is the half‐width at half maximum (HWHM) of the ODMR spectrum and is considered a constant during the simulated temperature range [2]. The theoretical maximum photon detection rate *R*_max_ is estimated by measuring the photon detection rate *R* as a function of the excitation laser power *I*. Considering the objective lens collection efficiency of *η*_col_≈0.04%, the fit to the saturation model *R*(*I*) = *η*_col_∙*R*_max_/(1+*I*_sat_/*I*) yields a saturation laser power *P*_sat_ = 0.22 ± 0.06 W and *R*_max_ = (2.7 ± 1.1)×10^14^ photons s^−1^, which are typical for NV ensemble magnetometry experiments [36, 37]. Note that in our experiment, the photon detection rate was attenuated to approximately 1.5×10^6^ photons s^−1^ to maintain the detector within its linear response range. The spin contrast *C*_cw_ in cw‐ODMR scheme is obtained from the simulation results of the *m_s_* = + 1 state in Figure 3b. In Equation (5), *S·∆*_1_​ governs the lattice relaxation energy associated with the ZFS shift, which steepens the slope *dω*_+_(*T*)/*dT*. While *ΔE_ms_*_= 0_ governs the ISC transition barrier, which limits the spin contrast C_cw_​. The energy matching between *∆E_ms_*_= 0_ and *S·∆*_1_​​ ensures that the enhancement in temperature sensitivity from the steepened ZFS shift effectively compensates for the ISC‐induced degradation in spin contrast, thereby preserving the temperature sensitivity* η*_cw,T_ at high temperatures. As *dω*_+_(*T*)/*dT* increases with temperature, *η*_cw,T_ improves from 57 µK Hz^−1/2^​ at 210 K to 8.3 µK Hz^−1/2^ at 490 K. At higher temperatures, the increased slope effectively compensates for the accelerated decrease in the spin contrast, stabilizing *η*_cw,T_ ​around 9 µK Hz^−1/2^ as shown in Figure 4a.

**FIGURE 4:**
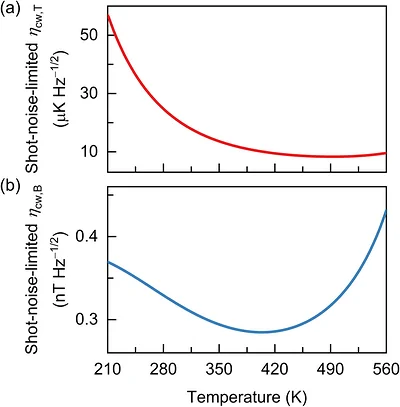
Temperature dependence of the shot‐noise‐limited (a) temperature and (b) magnetic field sensitivity.

Analogously, the shot‐noise‐limited magnetic field sensitivity *η*_cw,B_ is defined by [2, 31]

(6) $$\;\eta_{cw,B}=\frac{8\;\Delta \omega }{3\sqrt{3}\;\gamma_{NV}\;C_{cw}\;\sqrt{R_{max}}}\;.$$

Unlike *η*_cw,T_, *η*_cw,B_ mainly depends on the spin contrast C_cw_, not the ZFS shift. Therefore, the energy match does not directly compensate for *η*_cw,B_. As shown in Figure 4b, the temperature dependence of *η*_cw,B_ follows that of the spin contrast *C*_cw_ in Figure 3b, which first improves with increasing temperature to the optimum of 0.28 nT Hz^−1/2^​ at 404 K, then slightly degrades upon further heating. At the highest temperature in the simulation of 560 K, the shot‐noise‐limited temperature and magnetic field sensitivity remain as high as 9.6 µK Hz^−1/2^ and 0.43 nT Hz^−1/2^, respectively, highlighting the potential of the lifetime readout for high‐temperature precision quantum sensing.

Then, we experimentally measured the temperature sensitivity as well as the magnetic field sensitivity according to Reference [38]. At a given temperature *T*_ref_ as a reference, the lifetimes at three specific MW frequencies are selected to evaluate the sensitivity. *τ*_1_ and *τ*_2_ are at the maximum absolute slope max|∂*τ*/∂*ω*| on either side of the *m_s_* = +1 resonance *ω*_+_ in the ODMR spectrum. And *τ*_3_​ is the lifetime measured when the MW frequency is off‐resonant at 2 GHz. We define a function *F* to help estimate the temperature precision,

(7) $$F=\frac{\tau_{1}-\tau_{2}}{\tau_{1}+\tau_{2}-2\tau_{3}}.$$

Then the temperature precision is determined as

(8) $$\sigma_{T}\approx \sigma_{F}\frac{\Delta \omega }{\sqrt{3}\frac{d\omega_{+}\left(T\right)}{dT}},$$

where σ_F_ is the Allan deviation of *F*. σ_F_ is calculated according to 40 repeated lifetime measurements at each MW frequency. We measured σ_F,_ which is dependent on the effective integration time Δ*t* from 0.5 to 70 ms. Here, Δ*t* is the scaled actual measurement time according to the optical attenuation of 23 dB. We plotted *σ*_F_ as a function of both integration time and temperature in Figure 5a, and it decreases uniformly with integration time across the entire temperature range. The HWHM of the ODMR spectrum *∆ω* remains constant around 4 MHz throughout the experimental temperature tuning range. Benefiting from the robustness of the lifetime readout, σ_F_ tends to be stable upon heating. Meanwhile, as *dω*_+_(*T*)/*dT* is the same as *dD*(*T*)/*dT*, it increases with the temperature. As a result, an improved temperature precision could be observed at high temperatures in Figure 5b. Above 373 K, the long‐term temperature precision consistently remains as high as 0.3 K and is ultimately limited by the stability of the temperature controller, whose fluctuations are approximately 0.3 K at high temperatures. As the temperature sensitivity *η*_T_ is expressed by *σ*_T_ $\cdot \sqrt{\Delta t}$, there is an optimum for each temperature. As shown in Figure 5c, *η*_T_ reaches the highest value of 37 mK Hz^−1/2^ at 415 K, and remains higher than 55 mK Hz^−1/2^ at higher temperatures.

**FIGURE 5:**
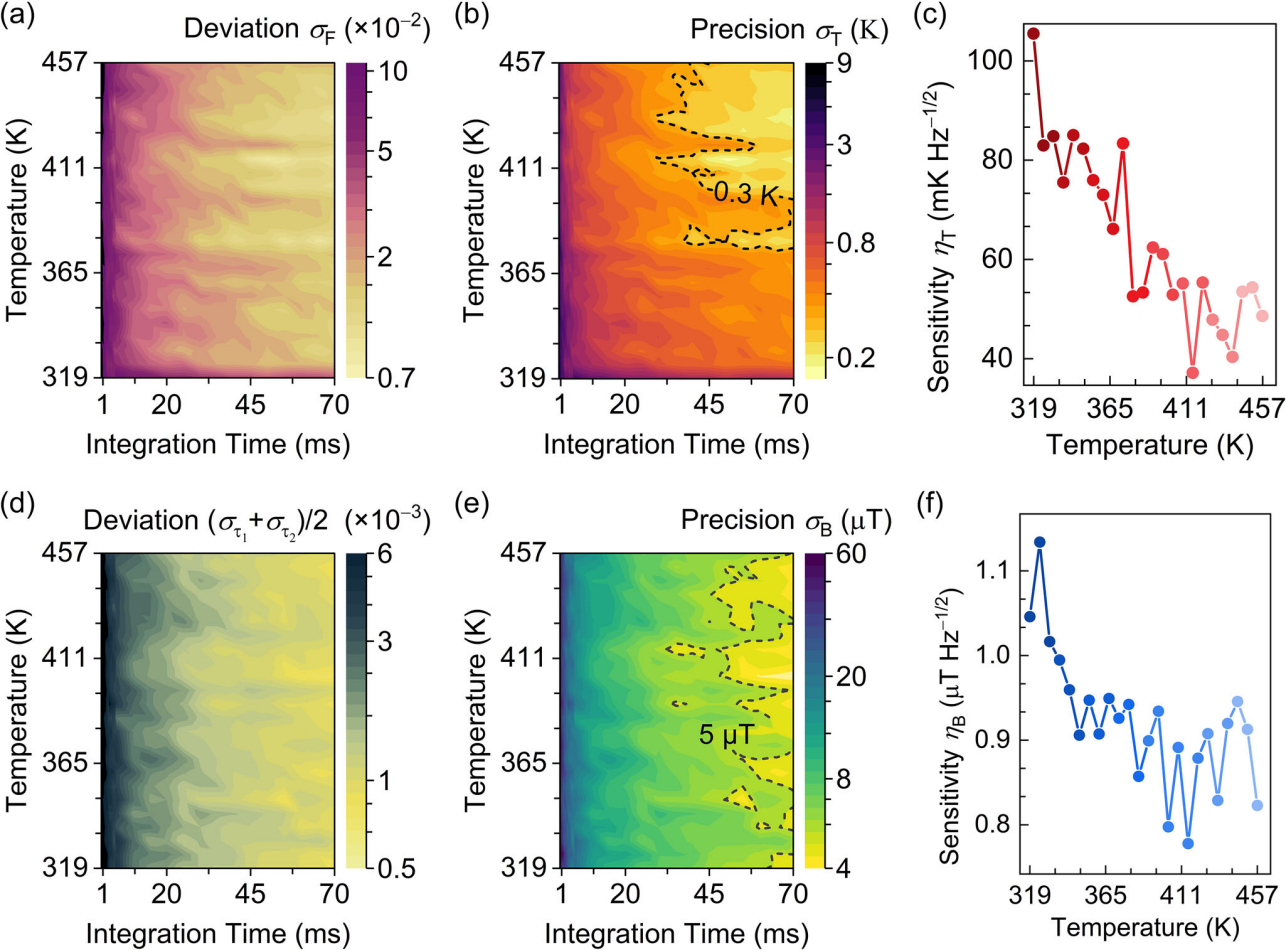
Effective integration time and temperature dependence of Allan deviation, sensing precision, and sensitivity in (a–c) temperature and (d–f) magnetic field, respectively. Dashed lines mark the position of precision isolines for *σ*_T_ = 0.3 K and *σ*_B_ = 5 µT.

In addition, the sensing of the magnetic field could be achieved by the lifetime‐based ODMR measurements at different temperatures. The magnetic field precision is calculated by [15]

(9) $$\sigma_{B}=\frac{1}{\gamma \;\cdot max\left|\frac{\partial \tau }{\partial \omega }\right|\;}\;\frac{\sigma_{\tau_{1}}+\sigma_{\tau_{2}}}{2},$$

where γ is the NV gyromagnetic ratio. *σ*_τ1_ and *σ*_τ2_ are the Allan deviations of *τ*_1_ and *τ*_2_, respectively. As shown in Figure 5d, the averaged Allan deviation (*σ*_τ1_+*σ*_τ2_)/2 exhibits a dependence on temperature and integration time similar to that of *σ*_F_, decreasing uniformly over the entire temperature range. The value of max|∂*τ*/∂*ω*| exhibits a temperature dependence similar to that of the spin contrast in Figure 3b. Correspondingly, the magnetic field sensitivity *η*_B_ = *σ*_B_ $\sqrt{\Delta t}$ first improves as the temperature increasing, reaching the highest of 0.78 µT Hz^−1/2^ at 415 K, and then slightly decreases. Above 415 K, the long‐term precision and sensitivity remain higher than 5 µT and 0.94 µT Hz^−1/2^ in Figure 5e,f, limited primarily by the degradation of spin contrast and the temperature fluctuation of the temperature controller.

Owing to the energy matching between the characteristic phonon modes governing ISC transition and ZFS shift, high‐temperature precision NV sensing via lifetime readout could be achieved. Given that the spin‐phonon interaction originates from the atomic displacements, this energy matching can be further understood within the configurational coordinate diagram, which links the equilibrium geometries of the relevant electronic states [30, 39]. Furthermore, by engineering the defect environment through concentration, strain, or isotopic composition, it is possible to design the matched phonon energies to suit specific thermal requirements. This framework can be extended to other solid‐state defect systems with analogous spin‐phonon interaction—such as silicon carbide vacancies or hexagonal boron nitride color centers [29, 40, 41, 42, 43]. Identifying and engineering the energy matching in spin‐phonon interaction provides a predictive framework for optimizing the sensing performance across diverse material platforms and operating conditions.

Beyond this mechanistic understanding, the high‐temperature sensing performance can be further improved by enhancing the steepness of the ZFS shift and suppressing the degradation of spin contrast. As several approaches have been demonstrated to enhance the NV spin‐phonon coupling in prior works [44, 45, 46], optimization of the strengthened phonon energy can maximize the temperature response of ZFS shift while minimizing the thermally induced spin mixing. Moreover, the lifetime readout directly captures the depopulation dynamics of the excited state. This capability is particularly valuable for probing the fundamental spin‐phonon interaction across a wide range of demanding conditions, such as the spin‐magnon‐phonon coupling in hybrid quantum systems and the high‐order spin‐phonon relaxation dynamics [19, 47, 48], enabling reliable measurements in complex and fluctuating environments.

## 3 Conclusion

In conclusion, we have developed a comprehensive theoretical framework for describing the NV spin‐phonon interaction in the ISC transition and the ZFS shift across a wide temperature range. We report an energy matching of the characteristic phonon modes governing these two processes, which enables the preservation of the precision sensing performance of the NV sensor at high temperatures. Thanks to the lifetime readout, we reach a shot‐noise‐limited temperature sensitivity of 8.3 µK Hz^−1/2^ at 490 K in the cw‐ODMR measurements of the NV ensemble. This work establishes a benchmark for the lifetime‐based sensing at high temperatures and reveals its potential in practical applications. The lifetime readout method can be extended to other solid‐state quantum platforms, providing a universally applicable strategy for characterizing spin‐phonon interaction and optimizing sensing performance.

## Author Contributions

**Yujing Cao**: investigation, writing – original draft, writing – review editing, formalanalysis, data curation, methodology, visualization. **Junjie Lin**: investigation, writing – review editing, software. **Wei Wang**: investigation, writing – review editing. **Qianru Chen**: investigation, writing – review editing. **Yan Liu**: investigation, conceptualization, writing – review editing, supervision. **Jie Ren**: writing – review editing, methodology, validation, formal analysis. **Heping Zeng**: funding acquisition, writing – review editing, project administration, resources, supervision, validation.

## Conflicts of Interest

The authors declare no conflicts of interest.

## Data Availability

The data that support the findings of this study are available from the corresponding author upon reasonable request.
